# Data, disease and diplomacy: GISAID's innovative contribution to global health

**DOI:** 10.1002/gch2.1018

**Published:** 2017-01-10

**Authors:** Stefan Elbe, Gemma Buckland‐Merrett

**Affiliations:** ^1^ Centre for Global Health Policy, School of Global Studies University of Sussex Brighton BN1 9SN UK; ^2^ Centre for Global Health Policy University of Sussex Brighton BN1 9SN UK

**Keywords:** data‐sharing, GISAID, global health, influenza, pandemic preparedness, public‐private partnerships, virus

## Abstract

The international sharing of virus data is critical for protecting populations against lethal infectious disease outbreaks. Scientists must rapidly share information to assess the nature of the threat and develop new medical countermeasures. Governments need the data to trace the extent of the outbreak, initiate public health responses, and coordinate access to medicines and vaccines. Recent outbreaks suggest, however, that the sharing of such data cannot be taken for granted – making the timely international exchange of virus data a vital global challenge. This article undertakes the first analysis of the Global Initiative on Sharing All Influenza Data as an innovative policy effort to promote the international sharing of genetic and associated influenza virus data. Based on more than 20 semi‐structured interviews conducted with key informants in the international community, coupled with analysis of a wide range of primary and secondary sources, the article finds that the Global Initiative on Sharing All Influenza Data contributes to global health in at least five ways: (1) collating the most complete repository of high‐quality influenza data in the world; (2) facilitating the rapid sharing of potentially pandemic virus information during recent outbreaks; (3) supporting the World Health Organization's biannual seasonal flu vaccine strain selection process; (4) developing informal mechanisms for conflict resolution around the sharing of virus data; and (5) building greater trust with several countries key to global pandemic preparedness.

## Impact Statement

The rapid spread of lethal infectious diseases is a global challenge potentially affecting any person around the world. To protect populations against such deadly outbreaks, it is critical that scientists and governments rapidly share information about the pathogens causing them. Without access to such information, it will be very difficult to properly assess the risk posed to global health, to develop new medical countermeasures, and to mount a commensurate international response. However, recent outbreaks suggest several impediments to the rapid sharing of virus data. Scientist may wish to withhold data until their scholarly studies are published; governments are fearful about the repercussions of being associated with a major new outbreak, and it remains challenging to fund global public goods like an international database to host such data. Through the first study of the Global Initiative on Sharing All Influenza Data (GISAID), this article shows how it is possible to encourage the greater international sharing of such data through the careful design of new sharing mechanisms. GISAID has now developed a successful track‐record in the field of influenza that may also serve as a useful blueprint for managing other diseases and global challenges requiring the international sharing of sensitive data.

## Introduction

**Impact Box**

What challenges does the study address?

The timely international sharing of virus data is critical for protecting populations against lethal infectious disease outbreak. Without access to such information, it is very difficult to properly assess the risk posed to global health, to develop new diagnostics, medicines and vaccines, and to mount a commensurate international response. However, experiences with recent outbreaks suggest that there are three challenges when it comes to sharing virus data. First, scientists may hesitate to share data on lethal viruses because they are concerned about other researchers then using this data to publish scholarly articles more quickly than they can do themselves – meaning their scientific contribution is not properly acknowledged and recognized. Second, governments might interfere with the international exchange of information because of concerns about the negative economic ramifications of being identified as the source country of an international outbreak. They may also wish to retain ownership over any potential intellectual property associated with the data and – particularly for low‐ and middle‐income countries – will be keen to ensure that they can secure access to new vaccines or medicines subsequently developed on the basis of that cooperation. Finally, there is also a more practical public goods challenge in terms of who will actually provide the funding and material infrastructure for hosting such virus data.

What is new about the research?

This research presents the first study of a new mechanism for encouraging the international sharing of virus data that has been created in the field of influenza. Initially spurred by the global threat posed by human infections with highly pathogenic avian influenza (H5N1), the Global Initiative on Sharing All Influenza Data (GISAID) was launched in 2008 as a new mechanism for incentivizing and promoting the international sharing of virus data.

What are the implications of the research?

The research shows how it is possible to overcome some of the challenges associated with the international sharing of virus data through the skillful design of new sharing mechanisms that are sensitive to the needs of stakeholders. Already, this important sharing mechanism has developed a successful track‐record in the field of influenza and may also serve as a useful blueprint for other diseases and global challenges that depend on the international sharing of sensitive data. The research further shows how philanthropic actors can play an important role in bringing about novel global health initiatives and how important it is to build trust in new global health initiatives. Finally, the research also illustrates how innovative solutions to global challenges can be found when lessons are creatively applied from one issue area to another and that such cross‐sectoral learning should be encouraged.

The rapid spread of lethal infectious diseases is a global challenge potentially affecting any person living around the world. Already on multiple occasions in the 21st century, a deadly new infectious disease emerged suddenly and then quickly spread through the dense network of international circulations that make up our globalized world – from HIV/AIDS and SARS, through pandemic flu and MERS, to recent experiences with Ebola and now the Zika virus. 1“Benefits of sharing”. *Nature*. 11 February 2016, p. 129. Sharing information on the viruses that cause such outbreaks is critical to protecting global health. Scientists need to rapidly share their information with other scientists around the world to understand the nature of the threat and to develop new medical countermeasures. Governments need the data to trace the extent of the outbreak, to coordinate public health interventions, and to ensure that populations have access to medicines and vaccines. All of this is particularly important in the case of flu, because of the comparatively rapid rate at which influenza viruses change, and the lurking spectre of a potentially devastating human pandemic. However, recent infectious disease outbreaks suggest that such international cooperation cannot be taken for granted and point to three impediments potentially hampering the timely sharing of such critical virus data.

First, scientists may hesitate to share data on lethal viruses because they are concerned about other researchers using this data to publish scholarly articles more quickly than they themselves are able to – meaning that their scientific contributions would not be properly acknowledged and recognized. Second, governments might also interfere with the international exchange of information because of concerns about the negative economic ramifications of being identified as the source country for an international outbreak. They may also wish to retain ownership over any intellectual property potentially residing in such data and – particularly for low‐ and middle‐income countries – will wish to ensure that they can secure access to new vaccines or medicines developed on the basis of that cooperation. Finally, even where these two challenges can be overcome, there is still a much more practical obstacle in terms of who will actually provide the international leadership, legitimacy, coordination, and funding needed for sustaining the material infrastructures essential for collecting, curating, and distributing such virus data. The international sharing of virus data may be critical to global health, but it is also enveloped in a Gordian knot of complex policy challenges.

The Global Initiative on Sharing All Influenza Data is an initiative aimed at untying that knot. Initially spurred by the global threat posed by human infections with highly pathogenic avian influenza (H5N1), GISAID introduced a new mechanism for incentivizing and promoting the international sharing of influenza virus data. GISAID's pivotal innovation consists of governing access to the data through a unique data access agreement extending a number of key “protections” and assurances to data contributors. In order to access the new database, users would first have to positively identify themselves through an initial registration and log‐in process so that access to the data could be monitored. As part of that initial procedure, users would also agree to acknowledge those who submitted the data in their publications and to make best efforts to work collaboratively with data contributors on scientific publications. Users would further have to agree not to share the data with third parties outside the GISAID community and would also not seek to place any restrictions on the use of the data. Through extending such additional “protections,” it was hoped that the GISAID mechanism could actively incentivize scientists and governments around the world to share influenza virus data in a timelier manner.

As GISAID marks its tenth anniversary, this article undertakes the first in‐depth analysis of its wider contribution to global health. The primary source material for the analysis comes from more than 20 semistructured, background interviews conducted with key informants in the international community. Those stakeholders were drawn from the international scientific community, government institutions, pharmaceutical companies, research institutes, international organizations, and many of those involved in creating GISAID itself. Additional primary and secondary source material was identified in the form of policy papers, background papers, working papers, official documents, articles and books on virus sharing produced by science journalists, governments, think tanks, scientists, and international organizations. The article argues that GISAID is making at least five key contributions to global health: (1) collating the most complete repository of high‐quality influenza data in the world; (2) facilitating the rapid sharing of potentially pandemic virus information during recent outbreaks; (3) supporting the World Health Organization's biannual seasonal flu vaccine strain selection process; (4) developing informal mechanisms for conflict resolution; and (5) building greater trust with several low‐income and middle‐income countries key to pandemic preparedness.

## Obstacles to the international sharing of virus data

The international exchange of influenza virus data, including for viruses with human pandemic potential, is particularly important because influenza viruses evolve more rapidly than many other viruses. 2Interview with Philip Dormitzer, Vice President and CSO: Viral Vaccines, Pfizer Vaccine Research and Development. 24 March 2015. Influenza, in other words, is a fast moving target. A World Influenza Centre was thus established at the Medical Research Council's National Institute for Medical Research in London in 1948. The World Health Organization (WHO) subsequently assumed the coordinating role for influenza virological surveillance with the establishment of the Global Influenza Surveillance Network (GISN) in 1952 – renamed the Global Influenza Surveillance and Response System (GISRS) in 2011. 3Interview with Alan Hay, former Director of the WHO Collaborating Centre (World Influenza Centre) in London (1993 to 2009); Co‐Chair of Scientific Advisory Council and Scientific Liaison Officer of GISAID. 15 September 2014. The network gradually expanded over subsequent decades, with a small number of laboratories becoming designated core WHO Collaborating Centres and receiving specimens/viruses for analysis from currently approximately 140 WHO‐designated National Influenza Centres located in more than 100 countries around the world.

Much of this early influenza work was carried out on the basis of biological characteristics of the viruses. As genetic sequencing technology became more widely available over the past two decades, however, so too genetic sequence data became more central to the process. 4Interview with Alan Hay. At the same time, and with laboratories now sequencing more and more influenza viruses, it was also becoming evident that in practice much of these data were *not* being shared and *not* being made public. 5Interview with John McCauley, WHO Collaborating Centre for Reference and Research on Influenza, Crick Worldwide Influenza Centre. 9 February 2015. That is because there are at least three obstacles to the timely international sharing of influenza virus data.

### Science, publications and recognition

The intensely competitive nature of science is one reason why virus data may not be shared in a timely manner. In a context where the standing of scientists, and the research income they can generate, is heavily linked to their publications, citations, and scientific reputations, there is pressure to be the “first” to publish findings – especially about a lethal new virus. Several interviewees expressed the view that scientists are concerned that sharing such information in an open and timely manner might enable others to publish findings with their data more quickly than they themselves could – meaning that their scientific contribution in discovering and analysing a new virus would not be properly credited and acknowledged (Longo and Drazen, 2016; Pearson, 2006: 963). Indeed, a number of interviewees expressed concerns about other researchers who just “crunch” the data generated and made available by others, without contributing to the generation of such data themselves, 6Interview with Gwenaelle Dauphin, Animal Health Service, Animal Production and Health Division, Food and Agriculture Organization of the United Nations, 9 October 2015; Interview with Yuelong Shu, Director of WHO Collaborating Centre for Reference and Research on Influenza, Beijing. 4 June 2014; Interview with John McCauley. as well as the importance of end users giving appropriate credit to originators. 7Interview with Ian Brown, Director of EU/OIE/FAO International Reference Laboratory for Avian Influenza and Newcastle Disease. 16 September 2014; Interview with Alan Hay. Scientists from low‐income and middle‐income countries have also complained that analyses from samples they shared in the past (because they lacked the powerful molecular research capacity of laboratories in high‐income countries) have subsequently been presented at international meetings and conferences without proper advance notification, or without including those who had shared the samples in the authorship arrangements (Sedyaningsih et al., 2008). Historically, some scientists have therefore decided to share such virus information in public databases only *after* their scientific papers had been published – leading to delays in the international sharing of data to the potential detriment of global health. 8Interview with John McCauley. Interview with Nancy Cox, former Director of the Influenza Division, Centers for Disease Control and Prevention, Atlanta. 17 September 2014.


This obstacle to the timely sharing of influenza virus data became all the more disconcerting when, in 2003, lethal human infections with highly pathogenic avian influenza H5N1 viruses reemerged in Hong Kong. Those human deaths raised the specter of a potentially much more devastating pandemic, with the human mortality rate of the virus reported by WHO at around 60% (WHO, 2016). With so much concern, fear, and attention now centring on the lethal H5N1 virus, the possible reputational benefits to scientists from being the first to publish analyses of the viruses were all the greater. It is precisely during such high‐profile times that everyone wants to get there “first”. 9Interview with Alan Hay. At the same time, the scientists at the forefront of such new outbreaks also suddenly become extremely busy, as their laboratories have to go into overdrive and often do not have enough resources to meet this surge in demand, whilst the priority in these circumstances is to generate science and evidence in support of control programmes. 10Interview with Ian Brown, Director of EU/OIE/FAO International Reference Laboratory for Avian Influenza and Newcastle Disease. 16 September 2014. With an increased workload, scientists would have even less time than usual to write up their findings, meaning, there was a real risk that information critical to global health would not be shared quickly. Even though the need for sharing data is arguably much greater in the context of the threat of a human pandemic, so too are the obstacles to achieving such international sharing of virus data in practice.

### Governments, trade and access to medicines

Governments may have additional reasons for not wanting data about lethal viruses to be shared internationally. They could be concerned with the negative economic ramifications of being identified as the county at the source of a lethal new outbreak, 11Interview with Ilaria Capua, former Head of the Virology Department at Istituto Zooprofilattico Sperimentale delle Venezie, Padova. 22 June 2015. and do not wish to be seen as the country that “caused” a devastating human pandemic. 12Interview with Nancy Cox. There can also be intellectual property considerations surrounding such samples and information, which could be critical to the commercial development of new diagnostics, vaccines, and medicines. 13Interview with Philip Dormitzer; Interview with Ron Fouchier, Professor in Molecular Virology, Department of Viroscience, Erasmus MC Rotterdam. 15 September 2014. See also Mary Quick. ‘Non‐WHO global initiative on sharing avian influenza data’. *The Lancet Infectious Diseases* 6(10): 621. October 2006. Indeed, low‐income and middle‐income countries in particular will be concerned to ensure access to any new medicines and vaccines produced with the help of such samples and data – as new medical countermeasures may later turn out to be too costly or only available in insufficient quantities. 14Pearson, ‘Plan to pool bird‐flu data takes off’, p. 963.


Many of these issues came to a fore in 2006 when, amidst intense concern about a possible flu pandemic, media reports surfaced about critical H5N1 sequence data not being made freely accessible to all countries – raising issues around fairness in accessing such data (Brown, 2006). It also emerged that new pandemic vaccines were being developed on the basis of biological samples initially obtained from affected countries in southeast Asia, but that originating countries were not consulted over the subsequent movement and sharing of such viruses with third parties (especially with industry) – raising additional concerns about the transparency of the GISN sharing mechanism (Sedyaningsih et al., 2008). Later, it transpired that once new H5N1 vaccines had been developed, they were not economically viable for many of those affected countries that had initially shared samples. 15‘Statement by the Minister of Health of the Republic of Indonesia H. E. DR. Dr. Siti Fadilah Supari’. November 2007. Available at: http://www.ip‐watch.org/files/Indonesia_statement_WHO.pdf . [Accessed 11 January 2016]. Confronted with the prospect of having to ride out a flu pandemic without access to the same medical countermeasures available to many high‐income countries, governments of affected countries began to openly question whom the sharing of virus samples through the GISN actually benefitted.

All these issues culminated in a lengthy and acrimonious diplomatic dispute over international virus sharing. The dispute was triggered when Indonesia (at the time experiencing the highest number of human cases of H5N1 infection) unexpectedly decided to stop sharing “its” virus samples from 20 December 2006 – marking the start of protracted, high‐level diplomatic negotiations surrounding the equity, fairness, and transparency of influenza virus sharing (Supari, 2008: 24). In terms of global health security, Indonesia's decision was regarded as a potential disaster in the sense that WHO now did not have a complete picture of how H5N1 was spreading and evolving 16Interview with Robert Webster, Emeritus faculty, Department of Infectious Diseases St. Jude Children's Research Hospital, Memphis. 10 July 2015; see also Richard Holbrooke and Laurie Garrett, ‘'Sovereignty' That Risks Global Health’. *Washington Post*. 10 August 2008.; but it also exposed the deeper political sensitivities around the international sharing of virus samples that would need to be addressed. As genetic sequence data of influenza viruses was becoming increasingly central to pandemic preparedness efforts, similar sensitivities emerged around such data as well.

### Practical challenges: funding databases sustainably

There is also a much more practical obstacle to the sharing of virus data. Somebody needs to provide the international leadership, legitimacy, coordination, and funding necessary for maintaining the material infrastructures central to collecting, curating, and distributing such data. A database will require both a physical infrastructure, as well as scientific oversight to design the database, curate the information, liaise with laboratories around the world, and so forth. Yet several interviewees described just how difficult it is to secure funding for databases. They are not seen as glamorous or important as research projects, 17Interview with an expert in computational biology. 23 February 2015; Interview with a European expert in research and biotechnologies issues, formerly working in the European Commission. 19 February 2015. or indeed as an essential part of scientific intercourse that are central to advancement of scientific research. 18Interview with Alan Hay. Unlike funding for research projects, moreover, databases also require continuous and even indefinite funding commitments, which many funders can be loath to agree to. 19Interview with Catherine Macken, Bioinformatics Institute of the University of Auckland, New Zealand. 17. December 2015.


Nevertheless, such an influenza database was initially created in the USA, by the Los Alamos National Laboratories in New Mexico, with funding from the US Centers for Disease Prevention and Control (CDC). 20Interview with Nancy Cox. The database was created around the time of the first outbreak of lethal human infections with H5N1 in Hong Kong in 1997 and worked well for some years. 21Interview with Ilaria Capua. However, when H5N1 began to spread internationally in 2004, it became clear that a number of affected countries were very sensitive about sharing H5N1 virus information and did not want WHO to release the data to others without their permission (Roos, 2006). As a way of acknowledging and addressing such concerns, those overseeing the Los Alamos database decided to create a separate, password‐protected compartment from the seasonal flu database that would only be open to those working on sequencing H5 viruses. 22Interview with Nancy Cox.


Creating this private H5 compartment may have been a practical solution to the diplomatic sensitivities and tensions that were rapidly surfacing, but it also created new problems. Some scientists expressed frustration that without access to this compartment, they could not properly analyse how the viruses they were isolating related to the other viruses circulating internationally. 23Interview with Ron Fouchier. There was also a perception that the private compartment at Los Alamos was somewhat of a “club”, where only a limited group of scientists enjoyed access. 24Interview with Ilaria Capua. See also Steven Salzberg. ‘The Contents of the Syringe’. *Nature* 454. 10 July 2008, p. 161. One scientist to vocally draw public attention to this problem was the Italian veterinary scientist Ilaria Capua. In February 2006, her laboratory received a sample of an avian influenza virus infecting birds in Nigeria (Zamiska, 2006a). There was great scientific interest in the new sample because it was the first to come from Africa. Capua was thus offered access to the private compartment in return for sharing her findings with the Los Alamos database. 25Interview with Ilaria Capua. Capua, however, declined the offer and instead deposited the genetic sequence information in the public domain archive Genbank. Her decision to take a stand against that system, to challenge the status quo, and to invite other leading scientists to join her in pushing for a change of approach, attracted much scientific attention and media coverage at the time. 26Interview with Nancy Cox. With unfavourable media attention surrounding the Los Alamos database, combined with the lack of a sustainable funding mechanism, as well as decreasing support from the US CDC, there was now a need to find a new home where such influenza virus data could be shared. 27Interview with Masato Tashiro, former Director, WHO Collaborating Centre for Reference and Research on Influenza, Japan, 5 June 2014; Interview with Catherine Macken.


## A new Global Initiative on Sharing All Influenza Data (GISAID)

The idea for an improved way of promoting the international sharing of influenza virus sequences was initially discussed in 2006, with the call for a new global initiative on sharing avian flu data (Zamiska, 2006b). Eventually, becoming the Global Initiative on Sharing *All* Influenza Data, GISAID's genesis was closely associated with Peter Bogner – a studio executive with a background in creating and licensing media content, 28‘Rock'n Rollouts’. *Multichannel New International*. November/December 1995; Hans‐Juergen Jakobs, ‘Der V‐Faktor’, Sueddeutsche Zeitung. 10 January 2005. and in philanthropic behind‐the‐scenes work for organizations such as the United Nations and UNICEF. 29Peter Bogner was Co‐Chair of the UNICEF Entertainment Support Committee. Letter from Horst Cerni. 2. September 1988 Bogner provided the lion's share of funding for setting up GISAID (a low‐mid seven figure sum) and was key to the development of the licensing mechanism that defines the GISAID data sharing policy. 30Email correspondence with GISAID Initiative (Freunde von GISAID e.V.). 18. January 2016; Robin McDowell, ‘Indonesia agrees to hand bird flu information to new online database’. The Associated Press. 16 May 2008. As chief executive of its management board, he remains closely involved in the initiative to this day. 31Correspondence for the Meeting between German Government with GISAID. Bundesministerium fuer Ernaehrung und Landwirtschaft. 18. March 2015. Remarkably, Bogner had no background in influenza – or even global health more broadly – prior to his involvement in GISAID, although he did have extensive experience with media and global affairs more generally. In 2006, a number of events would begin to draw him more closely into the world of influenza.

Bogner's interest in pandemic preparedness was originally piqued during a breakfast with Michael Chertoff (then United States Secretary of Homeland Security) and a small group of executives at the World Economic Forum in Davos in January 2006. Not long thereafter, Bogner was also asked by the New York Office of the United Nations Secretary General to use his extensive media contacts to look into unfavourable media claims alleging that WHO was operating a “secret” database in Los Alamos for genetic sequences of highly pathogenic avian influenza (HPAI) viruses and first liaised with Dr. Margaret Chan, who would subsequently become Director General of the WHO. 32Email Correspondence between Margaret Chan and Peter Bogner. 17 March 2006. In April 2006, Bogner then attended a scientific influenza meeting in Cambridge where he met Nancy Cox, who that year became head of the influential influenza division at US CDC. Sharing a ride with Bogner back to London, Cox had an opportunity to elaborate upon the challenges surrounding pandemic preparedness and the importance of international influenza sample and data sharing (Zamiska, 2006b).

As Bogner became more understanding of the complexities involved, he also learned from Cox of Indonesia's dilemma surrounding a particularly disconcerting H5N1 virus outbreak in Karo, Sumatra – where limited human to human transmission could not be immediately ruled out. Given his political connections in Indonesia, Bogner was able to secure a meeting to speak directly with the Indonesian Minister of Health Siti Supari, who was leading Indonesia's approach to these issues. Eventually Bogner was even able to persuade Supari to share the sequences of the Karo cluster, resulting in their deposit in Genbank. 33CIDRAP, 'Indonesia, FAO, OIE pledge to publish H5N1 data'. 3 August 2006. Available at: http://www.cidrap.umn.edu/news‐perspective/2006/08/indonesia‐fao‐oie‐pledge‐publish‐h5n1‐data . [Accessed 11 January 2016]; Email correspondence between Peter Bogner and Nancy Cox. 3 August 2006; Email correspondence between John Sulston and Peter Bogner. 5 August 2006. It was an unexpected diplomatic breakthrough, and a decision quickly met with reciprocity by the US CDC, when Cox not long thereafter announced that they too would be making influenza data publically accessible (Quick, 2006: 621). By this time, Bogner had become deeply immersed in the issues around the international sharing of influenza virus data. 34McDowell, “Indonesia agrees to hand bird flu information”.


The creation of GISAID moved a step closer when the influential scientific journal *Nature* published a prominent letter signed by more than 70 leading scientists (including seven Nobel laureates) in August 2006. The letter – coauthored by Peter Bogner, Ilaria Capua, Nancy Cox, and David Lipman – proposed the creation of a new global consortium that would foster international sharing of avian influenza isolates and data (Bogner et al., 2006). The letter, whose signatures included many researchers and officials from countries directly affected by H5N1, stated the intention for scientists participating in the consortium to share their sequence data, to analyse findings jointly, and to publish results collaboratively (ibid.). Although initially only members of the consortium would be able to access the data, as soon as possible following analysis and validation (and no longer than six months later), the data would then be deposited in publicly available databases that are part of the International Sequence Database Collaboration (e.g. EMBL, DDBJ, and Genbank) (ibid.).

Bogner was prominently listed as first author of the *Nature* letter and coined the acronym GISAID. 35Interview with Peter Bogner, Chairman of GISAID (Freunde von GISAID e.V.). 28 November 2013. However, along with a number of other influenza scientists, he was also aware that notwithstanding its good intentions, the brief letter still lacked much practical detail, and that the core issues of transparency and equity of data sharing would likely remain unresolved if data archives with anonymous access to data (like Genbank) were used. Ultimately, they felt, the sharing of such data would only work if any rights to the data that may exist would remain untouched through the process of sharing. 36Ibid. Moving things forward in practice would thus necessitate much more extensive consultation with a range of different stakeholders and mediating the development of a new system satisfying their various needs and concerns. At this point, Bogner set out to use his considerable knowledge of media and licensing issues, along with a pool of legal experts in intellectual property, government lawyers, as well as the help of key influenza experts, to develop a new mechanism that would permit the sharing of data without delay in a publicly accessible and free database, yet to be developed. 37Ibid. Over the next 18 months, those efforts would focus on three key areas: (1) developing and negotiating with Members States the legal terms for a new database access agreement; (2) the technical design of a new influenza database; and (3) agreeing the initiative's governance structure.

### An innovative approach to data sharing: the GISAID data access agreement

How could a new system better incentivize the international sharing of virus data? One possible way forward would be to try and provide data contributors with additional protections and assurances about how their data would be used. This could be performed through the careful legal design of a new data agreement governing the submitters' deposits of, and users' access to, such influenza virus data. From late 2006 onwards, Bogner thus engaged a number of former colleagues and lawyers who had worked with him on intellectual property issues during his broadcasting days to help realize a new data licensing agreement. 38Ibid. Additional scientific input to this agreement, particularly in terms of providing the scientific language for the sharing agreement and helping to define the data, came from the Scientific Advisory Council (SAC). Constituted early on by GISAID, the SAC was initially chaired by Nancy Cox from the US CDC and is composed of fellow WHO Collaborating Centre directors and FAO/OIE Reference Laboratory counterparts, as well as established researchers in the fields of epidemiology, human virology, veterinary virology, and bioinformatics. The SAC is now co‐chaired by Nancy Cox and John McCauley of the Crick Institute.

Developing this new data access agreement (DAA) would require Bogner to travel extensively around the world in an effort to forge an international consensus on a novel sharing mechanism enabling public and animal health authorities to continue their surveillance work, that ensured manufacturers of medical countermeasures could continue their work on developing vaccines, antivirals, or diagnostic kits, and that also provided a transparent mechanism for researchers who had the publication of their manuscripts as their main focus. 39McDowell, ‘Indonesia agrees to hand bird flu information’. GISAID's resulting DAA, which came into force in May 2008, retained the principle of a publicly accessible database – meaning that any natural person (whether scientist or not) could obtain credentials to access data in GISAID, predicated upon a one‐time positive verification of the individual's identity, and agreement to the terms of the DAA, which license the use of data in GISAID. 40GISAID EpiFlu™ Database Access Agreement © 2008–2016. Freunde von GISAID e.V. Available at: http://gisaid.org/DAA. [Accessed 23 March 2016]. This process of positively identifying the contributors and users of data differs from the anonymous access afforded to public domain archives (like Genbank), but provides GISAID with the mechanism for enforcement, and makes users adhere to the rules set forth in the DAA. Further benefits of this system are that it makes it easier for scientists to discover and properly acknowledge those who contributed the data and to also assist with any biosecurity considerations that could potentially arise around the use of some such data. 41Email correspondence with GISAID Initiative (Freunde von GISAID e.V.). 26. September 2016. See also Lawrence O. Gostin, Alexandra Phelan, Michael A. Stoto, John D. Kraemer and Srinath Reddy. ‘Virus sharing, genetic sequencing, and global health security’. *Science*. 345(6202): 1295–1296.


The core provisions of the DAA thus include that users: (1) will share their own data and allow other users to access it; (2) that they will *not* share or distribute data submitted directly to the GISAID sharing mechanism to other non‐GISAID servers or to individuals/institutions who are not registered GISAID users; (3) that they will credit the use of others' data in publications; (4) that they will make best efforts to collaborate with the originating laboratory and involve them in analyses and further research involving the data; (5) that they will analyse findings jointly; and (6) that they will maintain common access to technology derived from the data so that it can be used not only for research but also for the development of medical interventions such as diagnostics, vaccines, or antivirals. According to the agreement, GISAID users thus have the right to develop a commercial product on the basis of data obtained through GISAID, but they may not impose any terms on the data itself (which remains the sole property of the contributor), and they must also seek to collaborate with the data contributors. 42Email correspondence with GISAID Initiative (Freunde von GISAID e.V.). 26. September 2016


Most crucially of all, and notwithstanding its status as a publically accessible database, GISAID would therefore *not* fall under the legal definition of “Public Domain”, because the GISAID license respects the ownership of data submissions by explicitly not permitting the removal – or waiving – of any potential pre‐existing “rights” to the data; to the extent that such rights might exist around the data, they would not be affected by virtue of them having been submitted to GISAID. 43Interview with Peter Bogner. The unique sharing mechanisms thus ensure that inherent rights (such as intellectual property rights) are not forfeit when sharing data. 44Statement of the Federal Republic of Germany. ‘On Substantive Issues and Concerns Regarding the PIP Framework and ITS Implementation’. Special Session of the PIP Advisory Group, 13 October 2015. In many ways, the successful development of this DAA offering additional legal protections and clarity would mark the key to GISAID's new virus sharing mechanism.

### EpiFlu™: creating a new influenza database

In parallel to the legal access agreement, it would also be necessary to develop the actual database itself – especially with the closing of the existing database at Los Alamos. Decisions would have to be made about where to physically locate the new database, how to design its structure and features, as well as making practical arrangements for its day‐to‐day running. In February 2007, it was announced that the GISAID initiative would collaborate with a Swiss consortium consisting of the Swiss Institute of Bioinformatics (SIB), and the Swiss bioinformatics company SmartGene. SmartGene would provide secure storage and analysis of the influenza data, whilst SIB would complement genetic sequence information with high‐quality protein annotation provided by the team of lead scientist Amos Bairoch, who was well known for having developed the Swiss‐Prot protein knowledge database. 45‘Swiss Consortium to Manage GISAID Database.’ GISAID/Swiss Institute of Bioinformatics/SmartGene Press Release. 19 February 2009; Email correspondence between Amos Bairoch and Peter Bogner. 27 September 2006. Those initial arrangements for a new database would later become embroiled in complex legal disputes around contractual obligations and the flow of public funds from the USA, via the World Health Organization. 46Robin McDowell, ‘Influenza Scientists, WHO face off in virus row’. The Associated Press. 3 October 2008. This resulted in GISAID establishing a new EpiFlu™ database in Germany in 2009, following the German government's proposal to ensure GISAID's continuation by acting as its new official host. 47‘Aigner unterstützt Ansiedlung einer internationalen Influenza‐Datenbank in Bonn’. German Federal Ministry of Food and Agriculture. 16 October 2009. Available at: http://www.bmel.de/SharedDocs/Pressemitteilungen/2009/249‐Ai‐Influenza‐Datenbank%20Bonn.html [Accessed 23 March 2016]


To design the new database, GISAID used the same technical staff (today's Database Technical Group) composed of experts who held daily responsibilities for the sequencing activities at leading institutions such as the US' CDC, the National Institute for Medical Research in London, as well as the WHO Collaborating Centres in Beijing, Melbourne, and Tokyo, or who had worked on the design of the now defunct Los Alamos Influenza Database (Schnirring, 2009). The new EpiFlu™ database in Germany was then developed by the Max Planck Institut for Informatics (in Saarbruecken) in consultation with the wider scientific community. 48‘GISAID Launches Second Influenza Database’. GISAID Foundation Press Release. 14 September 2009. Available at: http://www.prlog.org/10340963‐gisaid‐launches‐second‐influenza‐database.html. [Accessed 13 January 2016]. Because the data that had been uploaded into the original EpiFlu™ database still belonged to those who supplied it (rather than to SIB), the data could then be migrated to the new database in Germany following the consent of the original contributors (Greenemeier, 2009).

With the move to Germany, responsibility for hosting the EpiFlu™ database and GISAID platform would henceforth rest with the German government. 49CIDRAP, 'German government to host flu database', 20 October 2009 (http://www.cidrap.umn.edu/news‐perspective/2009/10/german‐government‐host‐flu‐database) [Accessed 07 January 2016]. Four German institutions in particular would play central roles: (1) the Federal Ministry of Food and Agriculture representing Germany; (2) the Friedrich Loeffler Institute (Germany's Federal Research Institute for Animal Health) responsible for data quality; (3) the Federal Office for Agriculture and Food for the technical hosting of the database; and (4) the Max Planck‐Institute for Informatics, which would develop a new software application. In 2011, the Federal Republic of Germany and GISAID also announced that the German government would be the long‐term host of the EpiFlu™ database and GISAID platform, which it continues to do to this day. 50German Federal Ministry of Food and Agriculture. (http://www.bmel.de/EN/Ministry/Research‐Innovation/_Texte/Influenza‐Database‐EpiFlu.html) [Accessed 07 January 2016]. Ensuring the proper design, implementation, and sustainability of the new EpiFlu™ database thus marked a second key dimension in GISAID's data sharing mechanism.

### Developing a governance structure

Establishing an appropriate governance structure formed the final element. Given the scientific and political sensitivities surrounding influenza virus data, a proper governance structure would be vital to ensuring the legitimacy, scientific credibility, independence, and sustainability of the new initiative. Indeed, without such a structure, it would be difficult to build the requisite level of trust amongst scientists and governments necessary for them to agree to share the data with the new initiative. GISAID's governance structure would thus come to comprise of three independently operating bodies: (1) a board of trustees charged with securing the independence of GISAID from political or commercial interests; (2) the Scientific Advisory Council, providing scientific inputs and oversight of the initiative; and (3) the Database Technical Group, offering expertise in developing the database. 51GISAID, “Publically accessible EpiFlu database featuring the world's most complete collection of Influenza data” (http://www.ble.de/SharedDocs/Downloads/06_Dienstleistungen/07_DienstleistungszentrumIT/100420_GISAID‐Flyer.pdf?__blob = publicationFile) [Accessed 30 November 2015].


In the end, then, it took a good year and a half to move from the initial aspiration for a new global consortium expressed in the 2006 *Nature* letter, to finalizing all of the careful legal, practical, and governance arrangements needed for launching a new virus data sharing platform. Getting the GISAID sharing mechanism off the ground ended up being much more than just a technical challenge of developing a new influenza database. Although the data and database remain central to the enterprise, GISAID represents a much wider international initiative comprising the EpiFlu™ database alongside its innovative sharing mechanism (enshrined it its database access agreement), as well as its wider governance structure. All three elements are critical to achieving its aim of actively promoting the timely international sharing of all influenza virus data. Once these elements were in place, GISAID could officially be launched in Geneva on the occasion of the 61st World Health Assembly in May 2008. 52GISAID, “Publicly accessible EpiFlu™ 2.0 Database featuring the world's most current and complete collection of Influenza Data”. Available at: http://www.influenza.spb.ru/files/GISAID_Flyer.pdf [Accessed 08 January 2016].


The initiative's activities in different countries (especially China, Indonesia, and the USA) were later streamlined by formalizing them into a registered nonprofit association in Germany operated exclusively for charitable, scientific, and educational purposes – called “Freunde von GISAID e.V. [Friends of GISAID]” 53GISAID, “Publically accessible EpiFlu database featuring the world's most complete collection of Influenza data” (http://www.ble.de/SharedDocs/Downloads/06_Dienstleistungen/07_DienstleistungszentrumIT/100420_GISAID‐Flyer.pdf?__blob = publicationFile) [Accessed 30 November 2015]; Correspondence with GISAID Initiative (Freunde von GISAID e.V.). 3 October 2016; Federal Ministry of Food and Agriculture. ‘BMEL unterstuetzt den Kampf gegen Influenzaviren’. Press Release. 5 November 2014. Available at: http://www.bmel.de/SharedDocs/Pressemitteilungen/2014/277‐KL‐GISAID.html. [Accessed 6 October 2016]. In 2013, the German government also reaffirmed its long‐term commitment to host the GISAID platform and the EpiFlu™ database, ensuring its sustainability. 54Correspondence with GISAID Initiative (Freunde von GISAID e.V.). 3 October 2016. Wider operations, including user management, bilateral consultations with member states and dialogue with international organizations, or scientific matters remain GISAID's responsibility. 55Cooperation Agreement between Freunde von GISAID e.V. [Friends of GISAID] and the Bundesministerium fuer Ernaehrung und Landwirtschaft [German Federal Ministry of Food and Agriculture]. 5 November 2014, p. 3. GISAID similarly handles registrations and technical support questions. 56Ibid. GISAID today also has other public‐private partnerships – with the US Centers for Disease Control & Prevention and Singapore's Agency for Science, Technology & Research – which contribute to the development of technology and the educational programmes of the initiative. 57Correspondence with GISAID Initiative (Freunde von GISAID e.V.). 3 October 2016.


## GISAID's contribution to global health

Has GISAID been successful in meeting its aims and objectives? As the initiative marks its 10th anniversary, there is substantial evidence of a sustained track‐record of successfully facilitating the international sharing of influenza virus data. Indeed, five contributions of GISAID to global health stand out: (1) collating the most comprehensive repository of influenza genetic sequences, as well as associated clinical and epidemiological metadata; (2) facilitating the rapid sharing of potentially pandemic virus data during recent outbreaks; (3) supporting the WHO's bi‐annual influenza vaccine virus recommendation; (4) developing informal mechanisms for conflict resolution around the sharing of virus data; and (5) building greater trust with many low‐ and middle‐income countries key to global pandemic preparedness.

### Comprehensive international influenza virus data

Since its formal launch GISAID has rapidly built up an international user base comprising more than 6500 users. 58Email correspondence with GISAID Initiative (Freunde von GISAID e.V.). 26. September 2016. Those users today span individuals at universities, research institutes and public health organizations, clinicians, animal health experts, bioinformaticians, epidemiologists, and members of industry from around the world. 59PIP Framework Advisory Group, ‘Draft optimal characteristics of an influenza genetic sequence data sharing system under the pip framework’. 2015. Available at: http://www.who.int/influenza/pip/advisory_group/draft_twg_doc.pdf . [Accessed 3 December 2015]. The fact that GISAID is now widely used by the WHO – comprising the WHO Collaborating Centres for Influenza, the world's National Influenza Centers, and others 60‘WHO Global Influenza Surveillance and Response System (GISRS)’. Available at: http://www.influenzacentre.org/centre_GISRS.htm. [Accessed 5 March 2016]. – has helped GISAID collate the most complete repository of high‐quality influenza data in the world. 61PIP Framework Advisory Group, ‘Draft optimal characteristics of an influenza genetic sequence data sharing system under the pip framework’. 2015. Available at: http://www.who.int/influenza/pip/advisory_group/draft_twg_doc.pdf . [Accessed 3 December 2015]. Influenza data curated by a combination of automatic and manual steps from more than 850 institutions are now held and governed by the GISAID database access agreement. 62Email correspondence with GISAID Initiative (Freunde von GISAID e.V.). 26. September 2016.


The EpiFlu™ database today contains the genetic sequences of more than 1,000 influenza viruses with Human Pandemic Potential (IVHPP). 63The GISAID Initiative website (http://gisaid.org) [Accessed 11 January 2016]; PIP Framework Advisory Group, ‘Draft optimal characteristics of an influenza genetic sequence data sharing system under the pip framework’. 2015. Available at: http://www.who.int/influenza/pip/advisory_group/draft_twg_doc.pdf. [Accessed 3 December 2015]. The most recent human influenza sequences – human isolates of the subtypes H5N1, H6N1, H7N3, H7N7, H7N9, H9N2, H10N8, and H3N2 – are all contained in the database. 64GISAID, “EpiFlu™ database features and tools presentation” WHO‐GISAID training workshop, St Petersburg 28–29 August, 2014, WHO‐GISAID training workshop. Available at: http://www.influenza.spb.ru/en/conferences/gisaid‐2014‐en [Accessed 30 November 2015]. The database also holds sequences from others hosts – with (as of 2014) humans making up approximately 69% of data, avian species 19%, and other mammals like swine 10%. 65Ibid. Geographically, data is received from around the world, with approximately 36% of submissions coming from Asia, 29% from North America, and 22% from Europe, 5% from Oceania, 4% from Africa and 4% from South America (as of 2014). 66Ibid. At the end of 2015, the EpiFlu™ database broke through the symbolic threshold of holding 500,000 genetic sequences. 67The GISAID Initiative website (http://gisaid.org) [Accessed 7 January 2016]. By October 2016, this number had risen to more than 650,000 genetic sequences. 68Search of EpiFlu Database performed on 4 October 2016.


Data directly submitted to the EpiFlu™ database are also regularly complemented with sequence data deposited into public domain archives that are part of the International Nucleotide Sequence Database Collaboration (covering DDBJ, EMBL‐EBI, and NCBI). Of the 172,322 virus isolates held in the EpiFlu™ database (as of 4 October 2016), around 40% (65,915) were submitted to GISAID directly, and approximately 60% (106,407) were initially uploaded through the International Nucleotide Sequence Database Collaboration (INSDC). 69Search of EpiFlu Database performed on 4 October 2016. The EpiFlu™ database thus holds both the sequence information submitted directly through the GISAID platform, as well as those submitted to INSDC databases. In light of GISAID's emphasis on hosting high‐quality data, the latter data go through a process of further curation before being incorporated into the EpiFlu™ database. Given how rapidly influenza viruses change, moreover, the ability of the GISAID sharing mechanism to attract recent data is particularly significant. Of the data contained in the EpiFlu™ database from viruses collected solely over the past year, 81% were submitted directly to GISAID – and 93% for viruses collected over the last 6 months. 70Search of EpiFlu Database performed on 4 October 2016.


Beyond genetic sequence data, the EpiFlu™ database also stores and provides around 50 different fields of associated metadata (of which most are searchable). 71Email correspondence with GISAID Initiative (Freunde von GISAID e.V.). 26. September 2016. Such metadata includes date of specimen collection, specimen source, date of virus harvest, antiviral susceptibility, and – for human samples – patient information such as age, gender, and health status. 72PIP Framework Advisory Group, ‘Draft optimal characteristics of an influenza genetic sequence data sharing system under the pip framework’. 2015. Available at: http://www.who.int/influenza/pip/advisory_group/draft_twg_doc.pdf. [Accessed 3 December 2015]. Along with a number of data analysis tools integrated into the GISAID platform, these additional data are seen by many researchers to be valuable features of the EpiFlu™ database, including for the purposes of surveillance and pandemic preparedness. 73Interview with animal health specialist. 16 September 2014 . At the time of writing, plans were also underway to launch the next‐generation of the database – EpiFlu™ 2.0 – with funding provided from 2013 to 2016 through the European Union's Research and Innovation funding programme. 74European Commission PREDEMICS Consortium. Available at: http://cordis.europa.eu/project/rcn/101795_de.html [Accessed 23 March 2016] According to the directors of the core WHO Collaborating Centres for Influenza, GISAID has rapidly emerged as an essential resource and an “irreplaceable cornerstone for public and animal health in the global fight against influenza” upon which the influenza community now depends. 75Letter of the Directors of the World Health Organization Collaborating Centres for Influenza to the German Government. 18 September 2015.


### Global health security: encouraging rapid data sharing during outbreaks

GISAID has also demonstrated its ability to promote the rapid sharing of virus data during several key outbreaks of wider concern to global health security. Indonesia's 2008 decision to resume sharing H5N1 data through GISAID was hailed as a diplomatic triumph. 76Interview with Masato Tashiro. GISAID would again play a key role in the 2009 outbreak of pandemic H1N1. In April 2009, the first news of the novel H1N1 virus initially threatened to overwhelm the database, yet registrations were maintained around the clock to ensure everyone had access to the data and could share early detection findings with health authorities. 77CIDRAP News, ‘Pandemic reveals strengths of new flu database’ (2009). Available at: http://www.cidrap.umn.edu/news‐perspective/2009/06/pandemic‐reveals‐strengths‐new‐flu‐database . [Accessed 26 November 2015]. By April 25, the US CDC had uploaded the first full genome sequence of the new virus from initial US cases into GISAID, instantly giving the research community its first detailed look at novel H1N1. 78Ibid. That information was also used for developing new diagnostics for the virus, 79‘Viral gene sequence to assist update diagnostics for swine influenza A(H1N1). Geneva: World Health Organization. 25 April 2009. as well as for subsequent attempts to develop new vaccines for pandemic H1N1. 80‘CDC Testing New H1N1 Vaccine Target’ Recombinomics Commentary 3 August 2011. Available at: http://www.recombinomics.com/News/08031101/H1N1_Q226R_Vaccine.html. [Accessed 3 March 2016]; and ‘WHO Testing New Pandemic H1N1 Vaccine Target’. Recombinomics Commentary 18 August 2011. Available at: http://www.recombinomics.com/News/08181101/H1N1_Vaccine_WHO.html. [Accessed 3 March 2016].


More recently, GISAID was again used for the rapid sharing of data about the potentially pandemic H7N9 virus that caused human deaths in China and therefore also raised significant international concern. China reported the H7N9 outbreak to the WHO on 31 March 2013, just 6 weeks after the first known person fell ill. 81ECDC, 'Human infection with a novel avian influenza A(H7N9) virus, China', 27 January 2014. Available at: http://ecdc.europa.eu/en/publications/Publications/influenza‐AH7N9‐China‐rapid‐risk‐assessment‐27‐January‐2014.pdf . [Accessed 11 January 2016]. On the same day, it published the genomic sequences of viruses from the three human cases then identified on the database of GISAID, along with sharing the data and live virus with the WHO GISRS and other laboratories. 82‘The fight against bird flu’ *Nature* (2013). Availabgle at: http://www.nature.com/news/the‐fight‐against‐bird‐flu‐1.12850 . [Accessed 25 November 2015]. Using the data, the new virus genes could be synthesized in the USA in a matter of days, thus enabling the vaccine company Novartis to also rapidly develop a new vaccine. 83Vaccine News Daily, 'Positive Phase I clinical trial results for Novartis H7N9 vaccine'. 19 November 2013. Available at: http://vaccinenewsdaily.com/stories/510535576‐positive‐phase‐i‐clinical‐trial‐results‐for‐novartis‐h7n9‐vaccine [Accessed 11 January 2016]. Since that time, other companies have also used data in GISAID to develop new H7N9 vaccines. 84‘Medicago Produces VLP Vaccine Candidate for Emerging H7N9 Virus’. *Global Biodefense*. Available at: http://globalbiodefense.com/2013/05/09/medicago‐produces‐vlp‐vaccine‐candidate‐for‐emerging‐h7n9‐virus/ [Accessed 3 March 2016]; ‘Vaxart develops H7N9 vaccine’. Vaccines News Daily. 28 June 2013. Available at: http://vaccinenewsdaily.com/stories/510534785‐vaxart‐develops‐h7n9‐vaccine. [Accessed 3 March 2016]. GISAID thus plays a crucial role in the timely exchange of information integral to the selection of pre‐pandemic vaccine viruses. 85Letter of the Directors of the World Health Organization Collaborating Centres for Influenza to the German Government. 18 September 2015.


### Strain selection for the seasonal flu vaccines

Beyond the specter of pandemic flu, EpiFlu™ also contains the most up‐to‐date collection of data on seasonal influenza viruses (Neher and Bedford, 2015). The EpiFlu™ database generally receives submissions of data from current/novel strains significantly quicker than data generated from retrospective studies (ibid.). The EpiFlu™ database is thus routinely used during the bi‐annual process of selecting viruses that will form the basis for the seasonal flu vaccine in the Northern and Southern Hemispheres. Data on seasonal human influenza viruses for the biannual vaccine strain consultation meetings (VCM) are deposited by WHO Collaborating Centres within a time‐frame of days to a few weeks of sequencing, depending on urgency and other circumstances. 86CIDRAP News, ‘Pandemic reveals strengths of new flu database’ (2009) Available at: http://www.cidrap.umn.edu/news‐perspective/2009/06/pandemic‐reveals‐strengths‐new‐flu‐database . [Accessed 26 November 2015]. Several of the WHO Collaborating Centres first used the database in September 2008 (following its earlier launch in May of that year) to make their recommendations for the Southern Hemisphere 2009 seasonal flu vaccine, and all of the centres subsequently used it in February 2009, to make recommendations for the Northern Hemisphere 2009–2010 vaccine. 87Ibid. Since that time, GISAID has been consistently relied upon by WHO Collaborating Centres for this selection process – giving the database simultaneous global health utility for pandemic *and* seasonal flu. 88Interview with Nancy Cox; ‘Virendatenbank GISAID – Global Initiative on Sharing All Influenza Data’. Max Planck Foundation. Available at: http://www.maxplanckfoerderstiftung.org/project/333/. [Accessed 23 March 2016].


### Resolving potential conflicts amongst stakeholders

Over the years, GISAID has also evolved a track record of successfully managing potential tensions and conflicts between stakeholders within the consortium. This became especially clear over the 2013 sharing of Chinese H7N9 virus data, which led to two sets of tensions. First, it emerged on 5 April 2013 that the vaccine company Novartis and the J. Craig Venter Institute were planning to use the sequences uploaded into GISAID to develop a new H7N9 vaccine with the support of the US CDC and with funding from the US government (Butler and Cyranoski, 2013). Because of time constraints and immense concern about the lethalality of the virus, however, they initially proceeded without involving the Chinese researchers. They, in turn, felt that this move was not in the spirit of GISAID's data access agreement, which required data users to make best efforts to collaborate with the originating laboratory responsible for obtaining the specimens. At this point, GISAID's president stepped in and was able to mitigate the situation because of his close ties with the parties involved. Following discussions between the parties, the Chinese communicated to GISAID that they were satisfied that Novartis and its partners were engaging with China in a collaborative effort, and the vaccine development plans were able to proceed. 89Email correspondence with GISAID Initiative (Freunde von GISAID e.V.). 26. September 2016. Bogner was later commended for his efforts in this matter by both the former Head of Research at Novartis, Philip Dormitzer, as well as the head of the Chinese National Influenza Centre, Yuelong Shu. 90Ibid.


A second source of tension emerged around the same time when the Chinese scientists submitted their first major scientific paper on H7N9, including analyses of the sequences, to the prestigious *New England Journal of Medicine*. The Chinese researchers learned that they might be scooped, as a major analysis of the H7N9 virus was already due to come out in the journal *Eurosurveillance* on 10 April, with Masato Tashiro (then director of the WHO's Collaborating Centre in Tokyo) as a co‐author. Tashiro claims a draft of the paper was sent to the Chinese researchers along with an offer of co‐authorship, which was declined. 91Interview with Yuelong Shu; D. Butler and D. Cyranoski, ‘Flu papers spark row over credit for data’, *Nature*, 497 (2013), pp. 14–15. Bogner again played a key role behind the scenes in brokering a solution by effectively raising concerns of scientific etiquette that could be amicably resolved. Tashiro was asked to delay publication until after the Chinese research publication on 11 April, and their publication was published later – albeit still on the same day (Butler and Cyranoski, 2013). Although the episode confirmed the continuing tensions that exist around the international sharing of virus data, it also showed that such tensions could be constructively managed within the framework and spirit of GISAID.

### Building trust internationally

Despite being a relative newcomer to the global health landscape, GISAID has already garnered a reputation for building trust and respect internationally. 92Interview with Yuelong Shu. The fact that a substantial proportion of the influenza sequences deposited in GISAID's database originate from Asia and Africa is no coincidence. 93PIP Framework Advisory Group, ‘Draft optimal characteristics of an influenza genetic sequence data sharing system under the pip framework’, (2015) Available at: http://www.who.int/influenza/pip/advisory_group/draft_twg_doc.pdf [Accessed 3 December 2015]; Interview with Ron Fouchier. GISAID gave many LMICs the feeling that their concerns matter and that users of GISAID are treated equally. 94Interview with a European expert in research and biotechnologies issues, formerly working in the European Commission. 19 February 2015; Interview with Hanns‐Christoph Eiden, President of the Federal Office for Agriculture and Food, Bonn. 20 February 2015. The list of countries that have submitted data thus includes laboratories located in Vietnam, Brazil, Argentina, Cambodia, Thailand, India, Chile, Kenya, and Morocco. 95Search of EpiFlu database performed on 7 January 2016. It also includes countries that are deemed, because of their geographical location and role in responding to past outbreaks, to be central to global pandemic preparedness – including Indonesia, Mexico, Egypt, and China (including Hong Kong). 96Search of EpiFlu database performed on 7 January 2016. Overall, 201 countries and territories around the world participate in GISAID. 97Email correspondence with GISAID Initiative (Freunde von GISAID e.V.). 8 January 2016.


This trust from many low‐income and middle‐income countries is further nurtured through international workshops instructing researchers from around the world how to work and analyse data generated from viruses isolated in their region. To this end, GISAID has partnered with a number of other organizations such as the WHO's GISRS, the Antiviral Group of the International Society for Influenza and Other Respiratory Viruses Diseases (ISIRV), the PREDEMICS Consortium, and the Tan Tock Seng Hospital to host workshops in Africa, Asia, Russia, Europe, and the USA. 98GISAID Training Workshops, https://isirv.org/site/images/stories/avg_documents/Cape_Town/AVG‐GISAID%20Workshop%20Report_OptionsVIII.pdf; https://isirv.org/site/images/conferences/NGS/NGS%20Workshop%20Programme.pdf, https://www.isirv.org/site/images/Report%20on%20GISAID%20Workshop%20HK.pdf, https://www.ttsh.com.sg/page.aspx?id = 7014, http://www.influenza.spb.ru/en/conferences/gisaid‐2014‐en. [All accessed 23 March 2016]. Those workshops help to build further trust (as well as capacity) around the international sharing of influenza virus information amongst researchers from low‐income and middle‐income countries.

GISAID has also enjoyed other forms of international recognition. A meeting of the Association of South East Asian Nations (ASEAN) recognized GISAID in 2009 for encouraging the sharing of influenza genetic data. 99CIDRAP News, ‘Pandemic reveals strengths of new flu database’ (2009) Available at: http://www.cidrap.umn.edu/news‐perspective/2009/06/pandemic‐reveals‐strengths‐new‐flu‐database [Accessed 26 November 2015]. Its role in moving towards greater transparency and access concerning influenza virus genetic sequence data was also recognized by the 64th World Health Assembly in 2011. 100Sixty‐Fourth World Health Assembly. Geneva, 16–24 May 2011. Resolutions and Decisions Annexes. Available at: http://apps.who.int/gb/ebwha/pdf_files/WHA64‐REC1/A64_REC1‐en.pdf. [Accessed 14 January 2016]. Keiji Fukuda, in his capacity as Assistant Director‐General for Health Security at the WHO at the time, described GISAID as a “critically important and technically advanced new platform” that “provides an important option for sharing genetic sequence and epidemiological data” (Fukuda, 2011). The hosting of the database and platform by the German government is also seen to provide an important component of the trust GISAID now enjoys in many countries (ibid.). A final, but critical, component to maintaining this trust is GISAID's declaration that – since its formation – neither the initiative, its management, nor its board members have received research support, investment interests, or performed any contract work for industry or other commercial entities. 101PIP Framework Advisory Group, ‘Draft optimal characteristics of an influenza genetic sequence data sharing system under the pip framework’, (2015) Available at: http://www.who.int/influenza/pip/advisory_group/draft_twg_doc.pdf . [Accessed 3 December 2015].


## Conclusion

GISAID may have had an unlikely birth as a new global health initiative – with an unusually strong role played by an energetic, influential, and dedicated philanthropist without a prior background in global health. As the initiative marks its 10th anniversary, however, it is evident that GISAID is now making significant contributions to global health. Five such contributions stand out: (1) collating the most complete repository of high‐quality influenza virus data; (2) facilitating the rapid sharing of potentially pandemic virus information during recent outbreaks; (3) informing the WHO's biannual seasonal flu vaccine strain selection process; (4) developing informal mechanisms for conflict resolution; and (5) building greater trust with low‐income and middle‐income countries key to pandemic preparedness. Indeed, an array of interviewees pointed out that there is now widely perceived merit in GISAID's formula for balancing the need for control *and* openness, as well as the way it seeks to reconcile the competing imperatives of science, public health, and business. 102Interview with Philip Dormitzer. A number of interviewees within and outside of GISAID variously felt that it is functioning well as a mechanism and proving its value, 103Interview with David Heymann, Centre for Global Health Security, Chatham House, 9 June 2015. that there is a clear need for it, 104Interview with Nancy Cox. that it is (successfully) contributing to changing habits around virus sharing, 105Interview with Alan Hay. and that it has now effectively become the “go‐to” source for influenza information, especially when new outbreaks happen. 106Interview with Philip Dormitzer.


That said, even today it remains hard to know just how much genetic sequence data are still *not* being shared. Without knowing how many influenza viruses are being sequenced internationally (and when they are being sequenced), it is simply impossible to tell. 107Interview with Ron Fouchier. Some countries evidently prefer to share influenza data through GISAID rather than through other platforms that do not provide contributors with the same levels of protection yet some interviewees also expressed suspicion that – especially in the area of animal health – there still is much information that is not being shared, and that some countries are still only sharing a small proportion of information. 108Interview with Gwenaelle Dauphin.


Although GISAID has developed a distinct ethos for sharing, moreover, there are also natural limits regarding its ability to ensure that the terms of its access agreement are adhered to. Limiting access – and even outright exclusion – of those who violate the terms of the access agreement remains a credible sanction, and one that has been used in the past. According to GISAID, the percentage of all active users whose access credentials to the GISAID platform have been revoked at the time of writing is around 0.16%. 109Email correspondence with GISAID Initiative (Freunde von GISAID e.V.). 18 January 2016. At present, however, GISAID is only able to trace who is accessing information, not whether people are passing this information on to others. 110Interview with Masato Tashiro. GISAID maintains that if such data subsequently surfaces, they do have means to prove someone has illegally obtained the data – meaning that data contributors who suspect violations could seek to pursue this through legal channels. 111Email correspondence with GISAID Initiative (Freunde von GISAID e.V.). 18 January 2016. Nor, of course, can there be ultimate guarantees that people will adhere to these rules when confronting all the pressures of a pandemic situation in future, although the initiative has now developed a successful track record of navigating such situations. 112Interview with John McCauley.


Looking forward, there are at least three challenges that GISAID will need to navigate over the medium to long term. One such longer‐term challenge mentioned by several respondents revolves around the future leadership of the initiative. On the one hand, the material sustainability of the initiative is ensured for the foreseeable future through vital support provided by the federal government of Germany. 113Interview with an expert in computational biology. 23 February 2015. On the other hand, it is evident that the personal investment of significant amounts of time, energy, expertise, skill, and commitment by its philanthropic champion has been central to the success of GISAID. 114Interview with Ron Fouchier; Interview with an expert in computational biology. 23 February 2015; Interview with John McCauley. The longer term question of who will continue to provide wider leadership and the championing of GISAID will have to be addressed. Towards this end, the German government recently called for the institutionalization of GISAID to ensure its longevity. 115Minutes from the Annual General Meeting between Germany and GISAID. 28 April 2015.


A second challenging area for GISAID are the ongoing negotiations around the Pandemic Influenza Preparedness (PIP) Framework. The PIP framework creates responsibilities for the sharing of biological samples of influenza viruses with pandemic potential as well as providing a mechanism – partially funded by industry contributions – for the provisions of benefits (like medicines and vaccines) to affected countries. The question of whether genetic sequences data (as opposed to physical specimens) should also be governed by the framework proved too sensitive to be resolved during the initial negotiations for the PIP framework (Gostin et al., 2014). At the time of writing, GISAID is thus having to navigate a complex and sensitive set of diplomatic negotiations around the future role of genetic sequence data in the framework, with potentially considerable ramifications for the future of the initiative.

Probably, the biggest question to arise from GISAID's success, however, is whether its sharing mechanism can be extended to also cover other viral diseases and stand for a wider paradigm shift in improving international data sharing. As we have already seen several times over the past decade, influenza is not the only potentially lethal infectious disease that the world confronts, and it remains to be seen whether GISAID has both the aspiration and capacity to expand into other lethal viral diseases – such as Ebola, MERS, West Nile Virus, and other zoonotic diseases. 116Interview with an expert in computational biology. 23 February 2015; Interview with Ron Fouchier. There are certainly signs that this data sharing problem also manifested itself during the more recent Ebola outbreak in West Africa, (Yozwiak et al., 2015) as well as the spread of Zika virus. 117‘Brazil Considers Reforming Biosecurity Law Amid Criticism’. Associated Press. 6 February 2016. Available at: http://www.ndtv.com/world‐news/brazil‐considers‐reforming‐biosecurity‐law‐amid‐criticism‐1274312. [Accessed 10 February 2016]. Although GISAID was approached to extend its scope to include Ebola viruses, GISAID's leadership felt at the time that this was beyond its capacity. Yet Ebola is clearly another disease that shows more generally that the spread of viruses and their husbandry systems do not align naturally with political boundaries. 118Interview with Ian Brown. Here too, as in a number of other lethal infectious diseases, protecting populations will require creative and sustained efforts to carefully reconcile data, disease, and diplomacy.

## Conflict of Interest

The authors have declared no conflict of interest.

## Author Statement

S. Elbe and G. Buckland‐Merrett jointly conceived and designed the study, developed the study protocol, collected the data, analysed the data, and prepared and approved the manuscript.
